# Supplementary material for: Explanatory models of stillbirth among bereaved parents in Afghanistan: Implications for stillbirth prevention
Source: PLOS Glob Public Health. 2023 Jun 21;3(6):e0001420. doi: 10.1371/journal.pgph.0001420 (PMC10284382; doi:10.1371/journal.pgph.0001420)
Supplement: Attachment — Submitted filename: 2023_03_20_responses to reviewer comments.docx [file pgph.0001420.s001.docx]

**Responses to Reviewer’s Comments**

Below we provide point by point responses to reviewer’s comments in blue text. The line numbers we refer to are for the track change version of the manuscript.

**Reviewer #1: Reviewers comment

This study is really intriguing and may help the people of Afghanistan prevent stillbirths. But I have some reservations about the methodology component that the Authors required to address.
1. The authors mentioned that in line 112’ we used a qualitative study design employing semi-structured, in-depth interviews to elicit the…Is that supposed to be an approach? Can you describe the kind of design that was used for this particular study? , we use pronouns such as “I” and “we”. This is acceptable when writing personal information, or a book. However, it is not common in academic writing thus Avoid ‘we ‘ from your manuscript**

***Response:*** Yes, the study design is an *exploratory* qualitative design using semi-structured interviews. We have added this clarification in the manuscript in the abstract (p2) and methods (p5) section. We did not use any other theoretical approach.

Using the ‘active voice’ is considered standard practice and expected in public health academic writing. Using pronouns such as “I” and “we” is the preferred grammatical style in describing qualitative studies because it provides a clearer indication of how and what and helps understand the context. We prefer to keep this writing style and seek the editors advice on this point.

**2. It is unclear in this manuscript how the sample size was adjusted by the authors. Can you explain line 127 and why you only take 21 women? Please provide more information about how the sample size for the in-depth interview was determined.**

***Response:***

We used purposive sampling to recruit women for the interview. Prior to beginning the study we estimated that we would need around 20-30 interviews of women and 10-15 fathers to obtain a wide range of views. However, being a qualitative study, we remained flexible and once in the field we continued interviews until no significant new information was emerging (data saturation) based on the discussions with interviewers and preliminary analysis of the data. This was the basis of our decision to stop interviews. For female elders we had hoped to recruit more respondents but were unable to in the time frame we had for data collection.This is standard methodological practice when collecting qualitative data, and there is not a focus on the number of participants.

We have added some text to the manuscript to provide some explanation about this.

*Added text p6, line 131-140:*

*We used several methods for recruitment of mothers who gave birth to a stillborn baby – firstly, we identified women either facility medical records and those with phone numbers available were contacted by telephone to invite them to participate; secondly, we received notification of stillbirths that occurred directly from healthcare providers at the health facilities included in our study; and thirdly, through the interviewers’ community networks. Once mothers were identified we recruited fathers through those women and also through the networks of our interviewers’. We attempted to include women who gave birth at home as well as in the health facility but could only recruit five women who gave birth at home.. In rural districts, Community Health Workers (CHWs) assisted with identifying participants including female elders however we were only able to recruit 3 female elders in the time frame available for the data collection.*

**3. "Interviews were done by three experienced Afghan qualitative interviewers and a foreign female public health researcher," it was written at line 135. Does engaging a foreign researcher improve the quality of the data? Did they comprehend the culture? Could you please explain this?**

***Response:*** The foreign researcher (the lead author) did not conduct the interviews with community level respondents, only with key informants (in the health facilities or the Ministry of Heath). However, that data from those interviews is not presented in this paper so we have removed this statement now from the paper. The foreign researcher was the lead investigator in this study, and it was their PhD research.

**4. How do you assure the trust worthiness of the data??? Can you include this?**

***Responses:*** To ensure the trust worthiness of our data we performed ‘source triangulation’ by including multiple respondent types (multiple perspectives) – mothers, fathers, female elders and community health workers. During the data collection and analysis we had frequent discussions and debriefing sessions with our local interviewers to discuss the data. We have added further clarification to the methods about this:

*Added text p6, Line 150-152*

“Following each interview, interviewers completed a debrief form to document non-verbal observations, the interview environment, any new topics that arose, and challenges faced. *This was followed by debriefing discussions with the study team members to discuss the data and findings.”*

**5. One of the main rigor standards used within the qualitative research paradigm is triangulation. Why did the authors choose to conduct this study using just one method of data collection? Could you give your response to this?**

***Response:*** As we mentioned in response to comment 4, instead of ensuring rigour through triangulation of methods we used source triangulation by interviewing mothers, fathers, elders and community level workers. For this type of study, it was not feasible to conduct focus group discussions (FGDs) given the sensitive nature of the topic and this was the first study on stillbirth in Afghanistan. We had initially included the option for FGDs in the protocol, but the Institutional Review Board in Afghanistan during ethical review of the study requested we do not do this.

**6. "We employed the core domains of Kleinman's sickness explanatory model," was stated at line 155. Can you clarify this further by including it in the introduction section?.**

**Response:** On p4 in the introduction we state that “The explanatory framework by Kleinman[7] proposes that when there are differing models of illness between patient and health provider, this affects adherence with medical advice and treatment outcomes.”

We also have detail about Kleinman’s model in the methods section under the data analysis section so it could be repetitive to also include it again in the introduction.

The methods section (under data analysis) includes much detail about Kleinman’s model so it could be repetitive to also include it again in the introduction. We have copied the text below for your reference:

p7, Line 168 (Methods):

*We applied the key domains of Kleinman’s illness explanatory model[**7**] to thematically group codes into broader categories within this framework. Kleinman’s framework comprises of several aspects of disease that make up an explanatory model including definition of the illness, perceived causes/aetiology, symptoms, course of sickness, consequences, and treatment. We focused on the latter four categories to analyse our data.*

**7. This paper need extensive linguistic editing.**

**Response:** Could the reviewer be more specific on this point to indicate sections of text that need editing. We have two native English speakers from Australia as co-authors (AC and CRG) who have reviewed the manuscript in detail and could not identify major concerns.

**Reviewer #2: Thank you for the opportunity to review this paper. The authors have conducted a qualitative analysis of data from respondents in Afghanistan in 2017 on perspectives, attitudes and practise related to stillbirth. Seen as a continuum of the author’s work, where they have published the key findings from this work in another paper, this research adds to community beliefs and perceptions on this topic and represents an important contribution to the literature on maternal health.
The paper is methodologically sound and clearly communicates the key messages in its current format. However, issues in places are highlighted, and I encourage the authors to review them.**

1. The paper demonstrates encouraging results from the community members who mostly explain stillbirth through a biomedical lens; moreover, despite the country having seen considerable conflict-related challenges that have affected the status of women in society, most responses demonstrated relatively progressive and supportive families where women are by and large taken care of. **However, the paper does identify its limitation, but most of the respondents are urban residents who have given birth in health facilities which does colour in most of their findings. Kindly provide this facet earlier in the paper in the data collection stage as the readers required better context to the results**

***Response:*** Thank you for highlighting this. We have mentioned this as a limitation in the discussion (p34 also copied below), but we have also added more detail earlier in the paper emphasising this.

Limitations (p34)

*A key limitation of our study is that data collection was confined to only one province in Afghanistan which is the most progressive and urbanised and* ***most of our respondents had given birth at a health facility.*** *We were also limited to rural districts that had good access to health facilities and medical care all of which may limit the generalisability of our findings*

2. Another related aspect that **affects the selection of the respondents is whether they were directly chosen from the community or through health system records. As the authors indicated that most respondents came from health system interaction, please clarify why the sampling did not or could not also collect data from those women who had home-based deliveries or who had chosen not to go to health facilities.** It is expected that there will be a difference in beliefs and practises between groups of women who had stillbirths at an institution compared to those that did not.

***Response:*** Thank you for this valid point. When we first embarked on the study, we hoped to include women that gave birth at the facility and at home. Identifying these women was challenging which was why we started recruitment through health facilities. We identified a few respondents that gave birth at home (5/21) mostly through community networks of our interviewers, however, it was very difficult to identify and recruit. The other challenge was that most women who have a stillborn also have complications during the birth at home and then travel to a facility. Finally, we were restricted to Kabul province, due to security and safety concerns, and as Kabul is mostly urban, with better access to health facilities. We acknowledge this as a limitation of our study (please see the limitations section).

**3. Please review table two and the theme of extrinsic factors; some of these can fit under biomedical explanations.**

**Response:** When we refer to extrinsic, we refer to factors that occur externally or are related to actions or behaviour of others whereas biomedical causes were more related to biological factors or illness. We have tried to take a broad approach to this, we understand that the extrinsic factors can also lead to biomedical causes so they appear related/similar. This is just one interpretation and we acknowledge that there will be others.

**4. Could the authors please explain how the five themes present as results in lines 175 match with Kleinmans’ four categories that the authors used to frame the analysis(lines 159-161)**

***Response:*** We have applied Kleinman’s explanatory domains to guide our analysis and have adapted these domains – not directly used them. We summarise in the table below how they relate to the explanatory domains we use in this manuscript. We added one domain that was not included in Kleinman’s explanatory model – Prevention practices – as this we felt was important as it helps to understand perceptions of causation.

In the methods we have added further explanation to clarify which we hope helps:

p7, line 171-175

We focused on the latter four categories to analyse our data ***and adapt Kleinman’s domains for stillbirth creating five categories – i) perceived causes, ii) symptoms, iii) prevention practices, iv) consequences and impacts and v) treatment seeking and coping mechanisms. Prevention practices is a category we have added that we feel provides important explanatory information.***

| **Manuscript explanatory domains adapted for stillbirth** | **Kleinman’s explanatory domains for disease causation** |
| --- | --- |
| *Not included in this manuscript* | 1. Definition of illness |
| 1. Perceived causes of stillbirth | 2. Etiolology |
| 2. Prevention practices for fetal loss | *Not included in Kleinman’s model* |
| 3. Symptoms preceding stillbirth | 3. Symptoms/course of sickness |
| 4. Consequences and impacts of stillbirth | 5. Consequences |
| 5. Treatment seeking and coping mechanisms after stillbirth | 6. Treatment |

**5. The authors have already shared that they have published another paper from this data set which they reference(13). However, it was not immediately clear to me how this body of work is distinct from their previous publication. Could the authors kindly clarify how this paper is distinct from the previous research findings.**

***Response:*** This paper is focused on the data pertaining to community perceptions of what causes stillbirth and how these beliefs relate to health behaviours and actions, what actions and behaviours are taken did during pregnancy to prevent a pregnancy loss. The previous publication examined how perceptions and experiences of stillbirth *affect the reporting and recording of data on stillbirth* – the focus was on what perceptions affect disclosure of stillbirth and the accurate recording of data pertaining to stillbirth. We also included how stillbirth was defined and understood in this paper.

**6. As part of the presentation of results, Table 3 could be reduced together, removed**

***Response:*** Thank you, when we consider the whole of the results we think that this table succinctly summarises the theme and data. We would prefer to keep it, but will be guided by the editor’s decision.

**7. One of the major implications of their research which I agree with the authors is that healthcare workers holding superstitious views about stillbirth and communicating it to patients is alarming. Kindly consider adding this view to the table on the recommendations in the conclusion discussion section.**

***Response:*** Thank you for this suggestion. We have now added this to the table on recommendations.

*Discussion*

*p31 Box 1: “Include stillbirth prevention and management in training curriculum for doctors, midwives, nurses and community-level workers.* ***Education should include information on the preventability of stillbirths to dispel myths and incorrect beliefs surrounding these deaths.”***
